# Human Papillomavirus in Endometrial Adenocarcinomas: Infectious Agent or a Mere “Passenger”?

**DOI:** 10.1155/2007/60549

**Published:** 2008-01-02

**Authors:** A. Giatromanolaki, E. Sivridis, D. Papazoglou, M. I. Koukourakis, E. Maltezos

**Affiliations:** ^1^Department of Pathology, Democritus University of Thrace Medical School, 68100 Alexandroupolis, Greece; ^2^Department of Radiotherapy/Oncology, Democritus University of Thrace Medical School, 68100 Alexandroupolis, Greece; ^3^Second Department of Internal Medicine, Democritus University of Thrace Medical School, 68100 Alexandroupolis, Greece

## Abstract

*Aims*. To investigate the possible association of human papillomavirus (HPV) with endometrial hyperplasias and neoplasia. Does HPV play any role in the initiation or prognosis of endometrial adenocarcinomas? *Methods*. Twenty-five endometrial adenocarcinomas of the endometrioid cell type, with and without squamous differentiation, and twenty-four endometrial hyperplasias of various forms (simple, complex, and atypical) were analyzed for the presence of type 16 and 18 HPV by the polymerase chain reaction (PCR). The results were related to histopathological features of the tumour, and the patients' age, and prognosis. *Results*. Six of 25 endometrial adenocarcinomas were HPV 16-positive (24%), and 5 of 25 (20%) were HPV 18-positive. Simple endometrial hyperplasias was associated somewhat more commonly with HPV 16 and 18 (2/8 and 1/8 cases, resp.) than hyperplasias progressing to endometrial adenocarcinomas, namely, atypical endometrial hyperplasia (1/8 and 0/8 cases, resp.). None of the positive cases in the series, whether hyperplastic or neoplastic, demonstrated cytological evidence of HPV infection. There was no relation between HPV-positive cases and squamous differentiation, depth of myometrial invasion, lymphatic involvement, lymphocytic response, patients' age, or prognosis. *Conclusion*. It appears that the presence of HPV in the endometrium, as detected by PCR, does not play any role in the initiation or prognosis of endometrial adenocarcinoma.

## 1. INTRODUCTION

Human papillomavirus (HPV) infection in the female genital tract has been connected with specific sites: vulva, vagina, cervix, and specific epithelium—the stratified squamous epithelium, leading to epithelial cell proliferation and often malignancy. The infection, most reliably detected by polymerase chain reaction (PCR), is recognized by distinct histological changes in epithelial cells consisting of multinucleation and koilocytosis [1, 2]. Typical examples of the hyperplastic process include condylomata accuminata, and those of the neoplastic change include the intraepithelial neoplasias and the squamous cell carcinomas of the vulva, vagina, and cervix. In this context, HPV-infected cells have almost equated with frankly malignant cells and, indeed, the Bethesda system incorporated koilocytotic atypia and cervical intraepithelial neoplasia grade I (CIN I) into one category, the low-grade squamous intraepithelial lesions (LSIL), leading perhaps to excisional cone biopsy [3].

A number of reports have demonstrated the presence of HPV in endometrial adenocarcinomas [4–8], a tissue close to, but lacking, the stratified squamous epithelium of the exocervix. Whether such tumours with glandular-type epithelium may also exhibit morphological evidence of HPV infection is not clear, as koilocytotic-like changes have hitherto been only reported in the squamoid component of some endometrial adenocarcinomas [9, 10]. There is also some dispute as to whether the presence of HPV in endometrial tissues contributes to the development of endometrial neoplasms [6, 9, 11–13]. Thus far, we only know that HPV is unrelated to prognostic parameters and survival [7]; and there is, of course, little information with regard to the viral presence in endometrial hyperplasias [4, 8].

In view of this paucity of information, this study was designed to evaluate the presence of HPV in the endometrium by PCR and its potential role in the genesis of endometrial adenocarcinoma.

## 2. MATERIAL AND METHODS

The tissues used for this study were drawn from the files of the Department of Pathology, Democritus University of Thrace Medical School (Alexandroupolis, Greece). They were all hysterectomy specimens and had been routinely fixed in 10% formol-saline. The original haematoxylin and eosin- (H and E-) stained sections consisted of 24 endometrial hyperplasias of various forms: simple (n=8), complex (n=8), atypical (n=8), and 25 endometrial adenocarcinomas: endometrioid cell type, G1, stage I, with (n=5) and without (n=20) squamous differentiation.

The endometrioid adenocarcinomas and the various forms of endometrial hyperplasia were typed along the lines suggested by Buckley and Fox [14]. As G1 endometrioid adenocarcinomas were considered, only those composed in their entirety of glandular elements have no solid components, other than squamous, and no nuclear atypia, other than low grade [15]. The tumour stage was defined according to FIGO staging system [16]. None of the patients in the series had previous history of vulvar, vaginal, or cervical HPV-related lesion, intraepithelial neoplasia, or carcinoma. The duration of follow up was 5 years at least.

The tissues were analysed for the presence of type 16 and 18 HPV by PCR amplification (Laboratory of Second Department of Internal Medicine), Democritus University of Thrace Medical School (Alexandroupolis, Greece).

### 2.1. DNA extraction

Paraffin sections were cut at 7 μm and nonhyperplastic or nonmalignant tissue was trimmed away from the sample using parallel (H and E-) stained sections as a guide [17]. Normal sections were also cut and served as negative controls. Vigorous preparations were taken to avoid sample contamination. This was achieved by cleansing the microtome with 75% ethanol before and after cutting up each paraffin block, and using sterilized stainless forceps for transferring the sections. Paraffin wax was removed with xylene, and the samples were subsequently washed with 100% ethanol (the two steps repeated twice). DNA was isolated from the resuspended tissue using a commercially available kit, according to the manufacturer’s instructions (QIAamp DNA Mini Kit, QIAGEN Inc, Calif, USA). The human p53 and VEGF genes were used as controls to test the amplification ability of the extracted DNAs.

### 2.2. Detection of HPV

Isolated DNA was subjected to GP5+/GP6+ PCR [18]. Briefly, standard PCRs were carried out in 50 μL containing 50 mM KCl, 10 mM Tris HCL (pH 8.3), 200 μM each dNTP, 3.5 μM MgCl_2_, 1 U Platinum *Taq* Polymerase (Gibco, BRL, USA), and 50 pmol each of the GP5+ (5^'^-TTTGTTACTGTGGTAGATACTAC-3^'^) and GP6+ (5^'^-GAAAAATAAACTGTAAATCATATTC-3^'^) primers. Polymerase chain reaction amplification conditions were 96°C (1 minute), 45°C (1.5 minutes) and 72°C (1 minute) for 40 cycles, followed by final extension at 72°C for 10 minutes.

Single PCR was carried out using type-specific primers to investigate the incidence of both HPV 16 and HPV 18 sequences in GP5+/GP6+ samples (Cheng et al., 1995). The HPV 16 specific primers were as follows: Forward: 5^'^-CCCAGCTGTAATCATGCATGGAGA-3^'^ and Reverse: 3^'^-CACACGGGTAATTCAGAAGGT-5^'^ generating a 253 bps PCR product. The HPV 18 specific primers were as follows: Forward: 5^'^-CGACAGGAACGACTCCAACGA-3^'^ and Reverse: 3^'^-TCAATTTAGTAGTTGTAAATGGTCG-5^'^ generating a 201 bps PCR product. PCR amplification was carried out in 25 μL final volume containing 50 mM KCl, 10 mM Tris-HCl (pH8.3), 1.5 μM MgCl_2_, 200 μM each dNTP, and 1 U Platinum *Taq* Polymerase (Gibco, BRL, US). Polymerase chain reaction amplification conditions were 96°C (30 seconds), 60°C (30 seconds), and 72°C (30 seconds) for 35 cycles, followed by final extension at 72°C for 10 minutes.

The amplifications were carried out in a Mastercycler gradient (Eppendorf-Netheler- Hinz GmbH, Hamburg, Germany) thermal cycler and the PCR products were visualized in ethidium bromide stained agarose gels (2%).

In all PCR assays, appropriate positive controls for HPV 16 (human Caski cell line DNA), HPV 18 (human HeLA cell line DNA), and cervical squamous cell carcinomas were used and identified. In addition, the commercially provided positive controls for the identification of HPV 16 and HPV 18 by Maxim Biotech, Inc (San Francisco, Calif, USA) were applied successfully. Human lung and liver tissues were tested as negative controls and were consistently negative. All reactions were performed in a “blinded” manner by DP.

### 2.3. Statistics

Statistical analysis of the data was performed using Fisher’s exact test (SPSS, version 11.0.1).

## 3. RESULTS

Human papillomavirus (HPV) 16 was a somewhat more common inhabitant than HPV 18 in endometrial neoplasms and in all forms of endometrial hyperplasia. It was detected in 24% (versus 20% of HPV 18) of the endometrial adenocarcinomas studied, and in 16.6% (versus 4% of HPV 18) of the endometrial hyperplasias (Table 1). Both types of HPV occurred preferentially in simple endometrial hyperplasia rather than in complex or atypical hyperplasia, and there were three invasive endometrial neoplasms that were positive for both HPV 16 and 18. Interestingly, HPV were detected with approximately equal frequency in endometrial adenocarcinomas with squamous differentiation (1 in 5) and those without squamous elements (4-5 in 20) (Table 1).

**Figure 1:**
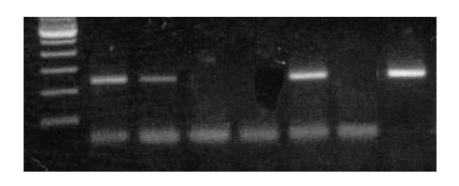
Agarose gel analysis of endometrial adenocarcinomas by PCR using HPV 16 type-specific primers. Line 1 (marker 100 bps), lines 2, 3, 6 (positive samples), lines 4, 5 (negative samples), line 7 (negative control), and line 8 (positive control).

**Figure 2:**
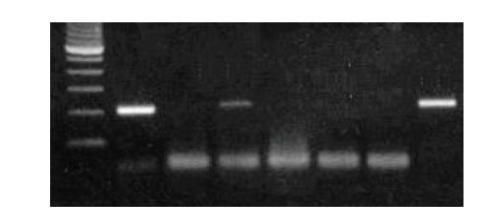
Agarose gel analysis of endometrial adenocarcinomas by PCR using HPV 18 type-specific primers. Line 1 (marker 100 bps), lines 2, 4 (positive samples), lines 3, 5, 6 (negative samples), line 7 (negative control), and line 8 (positive control).

Detection of HPV 16/18 DNA sequences in primary endometrial adenocarcinomas by PCR amplification products in representative specimens are shown in Figures 1 and 2, respectively.

None of the HPV 16 and 18-positive cases, whether hyperplastic or neoplastic, demonstrated cellular evidence of viral infection, that is, koilocytotic atypia or multinucleation. There was no statistical correlation between HPV 16/18 and squamous differentiation, depth of myometrial invasion, lymphatic involvement, lymphocytic response, patients’ age, or survival (data not shown).

## 4. DISCUSSION

The detection of human papillomavirus (HPV) in the human endometrium, whether hyperplastic or neoplastic, is fraught with curiosities. HPV was thought as being site- and tissue-specific, infecting the stratified squamous epithelium of the lower female genital tract: vulva, vagina, and exocervix [19], most commonly in connection with condylomata accuminata, intraepithelial neoplasias, and invasive carcinomas, and that the infected tissues suffered the cytopathic effect of multinucleation and koilocytotic atypia. Still, HPV 16 and 18, as detected by PCR, appear to reside in the endometrium, a simple or pseudostratified epithelium columnar in type, ciliated, in part, lining glands or glandular structures, and not having the characteristic cellular changes of HPV infection [1, 2]. Furthermore, the glandular lesions of the endocervix (adenocarcinoma in situ, invasive adenocarcinoma) may also harbour HPV 16 and 18 without morphological evidence of multinucleation or koilocytotic atypia [20–22].

Detecting HPV DNA sequences in tissues originally fixed in formaldehyde and embedded in paraffin wax may prove difficult by PCR methods [18], and the results obtained are inconsistent, as both specificity and sensitivity of various HPV PCR primer sets are not unaffected by intermethod variations [23]. This is reflected in the reported incidence of HPV 16/18 DNA detection in endometrial adenocarcinomas ranging from 4% to 37.5% [4–8]. Our results fall somewhere in the middle of this range (24% for HPV 16, and 20% for HPV 18) and are irrespective of the presence or otherwise of squamous differentiation.

Given that HPV infection precedes the development of cancer [24], it is also intriguing that HPV was detected less frequently in endometrial hyperplasias progressing to adenocarcinomas, namely atypical hyperplasia, than those not related to such development, namely simple endometrialhyperplasia, and in carcinoma. This apparently means that HPV cannot initiate oncogenic events in the endometrium through the sequence atypical hyperplasia-neoplasia. Similarly low or even lower, almost negligible, incidence of HPV infection for atypical hyperplasia were reported earlier [4, 8].

Interestingly, the presence of HPV in endometrial neoplasms was unrelated to histopathological features, patients’ age, or patients’ survival. This is also the experience of other investigators [8]. It is rather odd, however, that geographical/environmental conditions may influence the frequency of HPV detection; HPV 16 was detected in 6/47 (13%) endometrioid adenocarcinoma from Japan and 2/38 (5%) from the United States [4]. Others failed to detect HPV DNA in endometrial carcinomas [25–28], despite employing relatively large number of cases (66 in one study) and tumours with squamous cell elements (adenocarcinomas with squamous differentiation, squamous cell carcinomas) [26] or cervical tissues with stage II endometrial adenocarcinomas [28]; they suggested that the absence of HPV from the malignant endometrium is a hallmark of endometrial, as opposed to endocervical adenocarcinomas [28, 29].

Since HPV 16/18 infection is, by and large, site- and tissue-specific (vulva, vagina, and cervix stratified squamous epithelium), the endometrium, as indeed the glandular lesions of the endocervix, may not be a suitable host for HPV replication and maturation. This is further supported by the absence of relevant epithelial changes, lack of correlation with histological features or prognosis, and the low incidence rates with precancerous endometrial lesions. There is, of course, some evidence that koilocytotic-like changes may occur in the squamoid component of some endometrial adenocarcinomas with squamous differentiation [9, 10] and it is perhaps possible that at this site HPV positivity is preferentially present [30]. Nonetheless, the spread of HPV in the endometrium is not uncommon [4–8] and several cases in our material were positive for both HPV 16. We believe, as others do [9], that HPV, originated from the lower genital tract, represents a mere “passenger” in the endometrium, residing in its simple or pseudostratified columnar epithelium and having no aetiological or pathogenic role in the development of endometrial adenocarcinoma.

## Figures and Tables

**Table 1:** HPV detection in hyperplastic and neoplastic endometrium.

Endometrium	HPV 16	HPV 18	HPV 16 and 18
Simple hyperplasia	2/8 (25%)	1/8 (12.5%)	—
Complex hyperplasia	1/8 (12.5%)	0/8	—
Atypical hyperplasia	1/8 (12.5%)	0/8	—
Adenocarcinoma	3/25 (12%)	2/25 (8%)	3/25 (12%)
*Total adenocarcinomas*	*6/25 (24%)*	*5/25 (20%)*	—
